# At Europe’s migration crossroads: dialysis outcomes and transplant access in patients immigrating to Belgium

**DOI:** 10.1093/ckj/sfag096

**Published:** 2026-03-19

**Authors:** Lucas Jacobs, Maria Mesquita, Maxime Taghavi, Carine Verkest, Marianne Paesmans, Baptiste Dumoulin, Elena Vieru, Isabelle Brayer, Max Dratwa, Joëlle Nortier, Frédéric Collart

**Affiliations:** Nephrology, Brugmann University Hospital, Université libre de Bruxelles, Brussels, Belgium; Nephrology, Brugmann University Hospital, Université libre de Bruxelles, Brussels, Belgium; Nephrology, Brugmann University Hospital, Université libre de Bruxelles, Brussels, Belgium; Social Work Departments, Brugmann University Hospital, Université libre de Bruxelles, Brussels, Belgium; Clinical Research Unit, Brugmann University Hospital, Université libre de Bruxelles, Brussels, Belgium; Clinical Research Unit, Brugmann University Hospital, Université libre de Bruxelles, Brussels, Belgium; Nephrology, Brugmann University Hospital, Université libre de Bruxelles, Brussels, Belgium; Nephrology, Brugmann University Hospital, Université libre de Bruxelles, Brussels, Belgium; Nephrology, Brugmann University Hospital, Université libre de Bruxelles, Brussels, Belgium; Nephrology, Brugmann University Hospital, Université libre de Bruxelles, Brussels, Belgium; Nephrology, Brugmann University Hospital, Université libre de Bruxelles, Brussels, Belgium

**Keywords:** Belgium, ethnicity, immigrants, kidney transplantation, renal dialysis

## Abstract

**Background:**

Global inequities in dialysis and kidney transplantation among immigrant populations remain poorly characterized. At the crossroads of European migration, Brussels’ Brugmann University Hospital provides an opportunity to examine outcomes in one of the most ethnically diverse dialysis cohorts in Western Europe, including a substantial proportion of undocumented immigrants.

**Methods:**

We conducted a retrospective 15-year analysis of 497 incident dialysis patients (2010–2024), categorized as Western European (WE, 32%), Eastern European (EE, 21%), North African (NA, 26%), or Sub-Saharan African (SSA, 21%). Kaplan–Meier and Cox models assessed survival, while Fine–Gray competing-risk analyses evaluated transplant access.

**Results:**

Despite language barriers, housing instability, and the frequent lack of legal residency, equitable dialysis delivery was achieved, with peritoneal dialysis implemented in 25%–30% of patients, including asylum seekers and undocumented immigrants. Median pre-dialysis nephrology follow-up was longer in WE (8 months) than in other groups (<1 month). Age (HR = 1.73) and comorbidity (HR = 1.62) independently predicted mortality, whereas ethnicity (HR = 0.77) reflected demographic rather than systemic disparities. Competing-risk analysis showed the highest transplant access among SSA patients (35%–40% at 10 years; sub-distribution hazard ratios) = 2.13, *P* = .004), confirming that allocation was driven by clinical suitability rather than origin.

**Conclusion:**

In this uniquely multicultural setting, equitable access to dialysis and transplantation can be achieved across all immigrant groups, even among undocumented patients, but only when a dedicated hospital, social, and administrative structure is deliberately built to make it possible. The success of peritoneal dialysis among asylum seekers and the high transplant rates in SSA patients reflect an integrated system where equity is actively implemented, not assumed. These findings demonstrate that outcomes depend on clinical and organizational excellence rather than geography or migration status, offering a model for inclusive nephrology care in increasingly diverse European societies.

KEY LEARNING POINTS **What was known:**

- Immigrant and refugee patients often face major barriers to renal replacement therapy in Europe, with limited data on their dialysis outcomes and transplant access.

**This study adds:**

- In a multicultural Belgian dialysis cohort including asylum seekers and undocumented patients, equitable access to dialysis and transplantation was achieved through an integrated medical–social approach.
- Ethnicity predicted survival only through demographic and clinical factors, not healthcare inequities.

**Potential impact:**

- These findings show that organizational commitment and inclusive hospital structures can overcome social and administrative barriers, offering a model for equitable nephrology care in increasingly diverse European populations.

## INTRODUCTION

Chronic kidney disease (CKD) is recognized as an independent risk factor of cardiovascular diseases. As populations age, a stable age-standardized prevalence of CKD over the past three decades has led to an overall prevalence of 10%, along with increases in hypertension and diabetes mellitus [1, 2]. The growing burden of CKD is illustrated by its contribution to total mortality and its associated financial costs [3]. Although epidemiological studies exist on respective CKD stages, particularly end-stage kidney disease (ESKD), most of them focus on Western countries and their predominantly Caucasian population. Current global projections indicate that the largest absolute growth in ESKD cases will occur in developing regions, particularly Asia and Africa, due to demographic changes and rising prevalence of risk factors such as diabetes and hypertension [4].

Belgium holds a multicultural landscape in Western Europe, a third of its population being foreign or from foreign origin [5]. Beyond demographic diversity, several European studies have demonstrated that migrant populations face major barriers to equitable access to nephrology care and kidney replacement therapy (KRT). Migrants tend to initiate dialysis at a younger age, with significantly higher rates of emergency dialysis start, temporary vascular access use, and late referral to nephrology compared with native populations, and most remain unaware of their CKD at arrival [6].

In the field of kidney transplantation, first-generation immigrants born outside the European Union have a significantly lower probability of access to living-donor transplantation, while access to deceased-donor transplantation does not differ according to migratory status [7]. These disparities are mainly driven by linguistic, cultural, administrative and socioeconomic barriers, as well as insufficient access to culturally adapted education [8]. Our Nephrology and Dialysis Department is one of the largest centres in Brussels, public-funded, teaching, partnering federal and international agencies such as the Federal Agency for the Reception of Asylum Seekers (FEDASIL) or ‘Doctors of the World Belgium’, therefore welcoming economic, political, or health refugees from several countries immigrating into Belgium. By the end of 2024, our centre had 236 prevalent patients on dialysis, with more than two-thirds of non-Belgian origin coming from 32 different countries. Such demographic composition is increasingly representative of major European urban centres in the era of global migration, yet remains largely understudied in nephrology literature.

This study provides one of the first comprehensive analyses of survival and transplantation access in a highly diverse dialysis population within a WE setting. As multicultural populations increasingly shape healthcare needs, understanding clinical outcomes and access to renal replacement therapy in socially vulnerable groups is essential for planning and policy development. The aim of this study was 2-fold: first, to describe the demographic, clinical and socioeconomic characteristics of a uniquely diverse and socially vulnerable dialysis population treated in a large urban public hospital working in formal collaboration with social support organizations, a context that may influence patterns of care; and second, to compare dialysis initiation profiles, survival outcomes and access to kidney transplantation across WE, EE, NA, and SSA patients.

## MATERIALS AND METHODS

### Study setting

This study was carried out in the Nephrology Department of Brugmann University Hospital in Brussels, Belgium. We conducted a 15-year observational retrospective study on 497 incident patients starting dialysis treatment from 1January 2010 to the 31 December 2024 using the French-speaking Belgian Nephrologist Group (GNFB) database of ESKD.

The primary outcome was all-cause mortality. Secondary outcomes included kidney transplantation (incidence modelled with death as a competing event), emergency dialysis initiation, and dialysis modality at the start of chronic therapy.

### Inclusion and exclusion criteria

We included all incident ESKD patients starting a programme of renal replacement therapy (RRT) in our centre, thus excluding those who had been previously dialysed elsewhere. Patients requiring dialysis as a transient therapeutic modality and becoming free of any RRT due to recovery of kidney function, were excluded. Patients dying on acute dialysis treatment were excluded. Twelve patients originating from Asia or the Americas (<3% of the cohort) were not included in the analysis because they formed a very small and heterogeneous group, which did not allow meaningful analysis for this particular subgroup and would have reduced the stability and interpretability of the statistical models.

### Follow-up

Patients were followed from day 1 after the initiation of dialysis (baseline) until death or censoring (at time of first transplantation, lost to follow-up or permanent transfer to another dialysis centre). Incident dialysis modality represents the modality at start or after a brief period of urgent-dialysis modalities such as haemodialysis (HD) or continuous veno-venous hemo(dia)filtration (CVVH). Thus, incident HD represents patients starting long-term in-centre haemodialysis after completion of CVVH or acute HD, and incident Peritoneal dialysis (PD) represents patients initiating chronic peritoneal dialysis as their first stable modality. PD was never initiated as an emergency dialysis modality in our department. The observation period extended from 1 January 2010 to 31 December 2024, and all patients who had not experienced an event by that date were administratively censored at the end of the observation period.

### Populations and covariates

Patients were divided into four groups according to country of origin: Western Europe (WE; Belgium, France, Italy, Spain), Eastern Europe (EE; mainly Albania, Armenia, Poland, Romania, Ukraine), North Africa (NA; Morocco, Algeria), and Sub-Saharan Africa (SSA; Democratic Republic of Congo, Rwanda, Cameroon). Data collected included age, gender, primary renal disease, comorbidities, pre-dialysis nephrology follow-up, dialysis modality, survival, and time to transplantation (Table 1). Detailed individual administrative status (e.g. asylum seeker, undocumented migrant, temporary protection holder, regular resident) was not systematically recorded in the nephrology database and could therefore not be analysed as a separate variable. In practice, most EE, NA, and SSA patients were recent migrants managed either within the FEDASIL reception system or through the ‘Urgent Medical Aid’ scheme, whereas WE patients were predominantly long-term residents with standard health insurance.

**Table 1:** Basal characteristics of incident dialysis population from 2010 to 2024 at the nephrology department of Brugmann University Hospital.

	WE *N* = 161	EE *N* = 104	NA *N* = 128	SSA *N* = 104
Basal characteristics on arrival				
Male	96 (60%)	64 (61%)	74 (58%)	62 (60%)
Female	65 (40%)	40 (39%)	54 (42%)	42 (40%)
Mean age (years)	67	55	60	56
Mean Charlson score	4.2	3.8	3.7	3.7
Median months of follow-up before first dialysis (IQR)	8 (36)	<1 (17)	<1 (16)	< 1 (12)
Primary renal disease				
Renal vascular disease	41 (25%)	25 (24%)	20 (16%)	41 (39%)
Type II diabetes	52 (32%)	29 (28%)	57 (44%)	21 (20%)
Glomerulonephritis	5 (3%)	4 (4%)	1 (<1%)	3 (3%)
PKD	9 (6%)	8 (8%)	5 (4%)	0
Unknown	25 (15%)	16 (15%)	10 (8%)	15 (14%)
Other	29 (19%)	22 (21%)	35 (27%)	24 (23%)
Initial treatment modality				
Peritoneal dialysis	47 (30%)	25 (25%)	31 (25%)	33 (31%)
Haemodialysis	114 (70%)	79 (75%)	96 (75%)	71 (69%)
Mean dialysis vintage (months)	25.6	12.5	15.4	11.6
Mean time to transplantation (years)	1.7	5.3	3.5	3.6
Median survival time in months (IQR)	20 (40.7)	39 (57.4)	24 (23.1)	38 (50.2)
Outcome at censor day				
Still on dialysis	22 (14%)	19 (18%)	18 (14%)	24 (23%)
Deceased	74 (45%)	34 (33%)	36 (28%)	18 (17%)
Renal transplanted	24 (15%)	18 (17%)	15 (12%)	29 (28%)
Lost to follow-up	19 (12%)	21 (20%)	11 (9%)	11 (10%)

Primary renal disease was defined histologically when biopsy was available, or clinically otherwise. CKD aetiology was classified as: renal vascular disease in patients with longstanding hypertension and evidence of target-organ damage (left ventricular hypertrophy, retinopathy, or shrunken kidneys); diabetic kidney disease in diabetics with glomerular proteinuria and diabetic retinopathy; glomerulonephritis when documented or presenting as glomerular proteinuria/haematuria with hypertension without secondary causes; autosomal dominant polycystic kidney disease (PKD) by clinical or familial criteria. Rarer aetiologies (tubulointerstitial nephritis, cardiorenal syndromes, myeloma, congenital diseases) were grouped as ‘Other’, and unclassified cases as ‘Unknown’.

**Figure 1:**
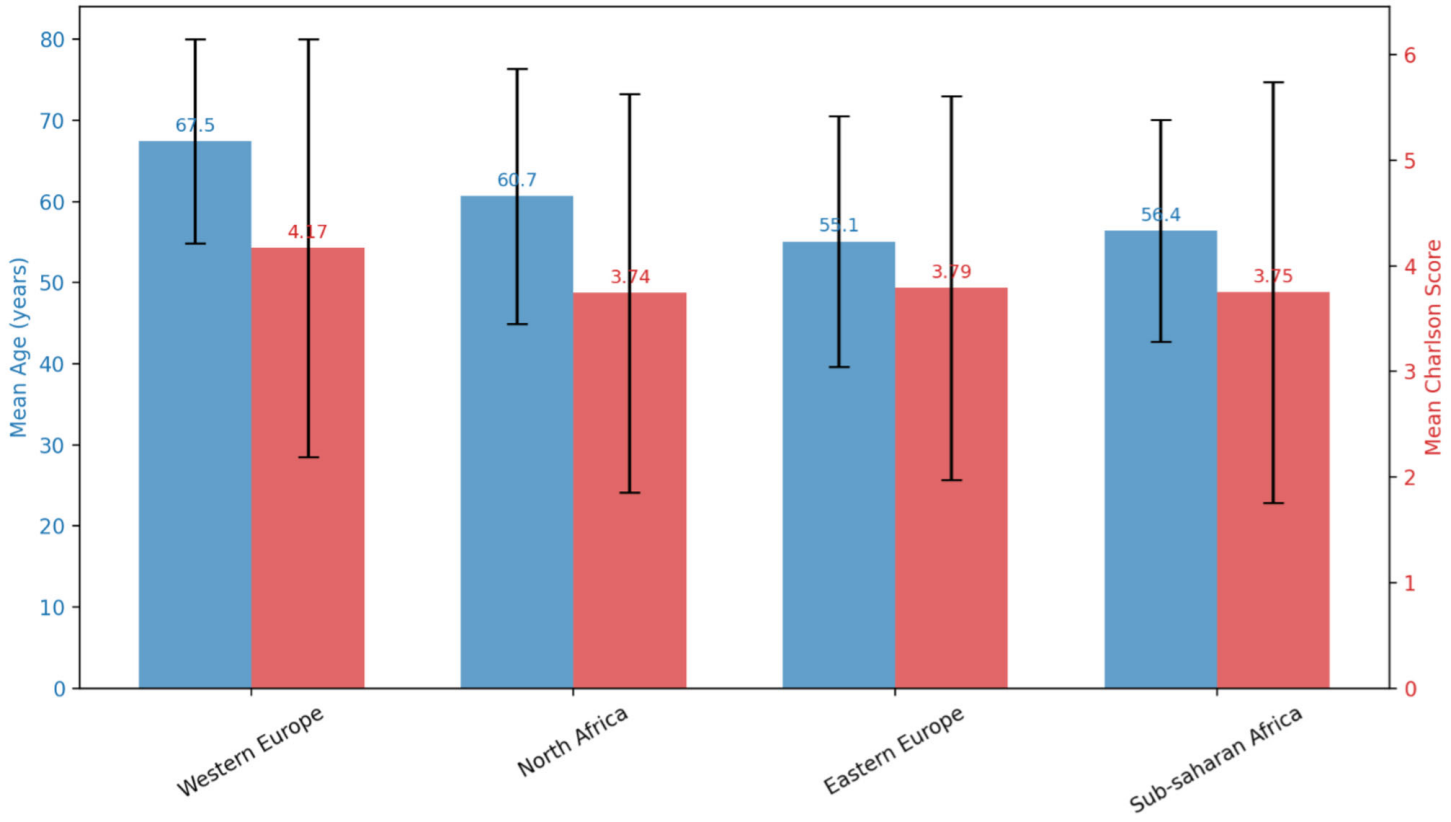
Overview of the variability of age and comorbidities (mean Charlson comorbidity score) between groups of patients immigrating from countries of different origin into the nephrology department of Brugmann University Hospital (Brussels, Belgium).

The Charlson comorbidity index (CCI) was calculated at dialysis start using 19 morbidity categories. [9, 10] As all patients had ESKD (2 points), scores were adjusted by subtracting these points (Fig. 1). Country of origin was derived from the GNFB database; for immigrants, it corresponded to the country of birth, and immigrant status reflected recency of migration and administrative situation at dialysis start.

Because our institution is not a transplant centre and transplanted patients are followed elsewhere, detailed information on donor type (living vs deceased donor) was not available, and donor type could therefore not be evaluated.

### Statistical analyses

The Mann–Whitney *U*-test was used to assess whether age and comorbidities significantly differed between groups. Chi square tests were used to compare groups for categorical variables.

Survival curves were estimated using the Kaplan–Meier method, and differences between groups were assessed with the log-rank test. Univariate analysis looking at the impact of the difference covariates was done. As all covariates we collected are identified in the literature as impacting on the survival, we then used a Cox regression model to estimate the impact of the ethnic origin adjusting for all of them (as we had a sufficient number of events, we did not use a directed acyclic graph to identify the minimally sufficient adjustment set). However, we also used a backward selection of covariates to get a more parsimonious model.

Access to transplantation across ethnic groups was analysed using competing risks methodology. We estimated non-parametric cumulative incidence functions (CIF) for transplantation with death as the competing event (Aalen–Johansen), and compared CIFs across groups using Gray’s test. We also fitted Fine–Gray sub-distribution hazard models to estimate sub-distribution hazard ratios (SHR) versus WEs, accounting for the competing risk of death. Because death prevents transplantation, it represents a true competing risk rather than censoring. The Fine–Gray model estimates SHR that account for this competing mortality, providing clinically relevant cumulative incidence estimates. Traditional Kaplan–Meier analysis would inappropriately consider deaths as censored observations, potentially overestimating transplantation probabilities and obscuring true access disparities between ethnic groups.

We considered a *P* value <.05 to be statistically significant and confidence intervals are reported using a 95% confidence level.

### Comparison

Our data were compared with those collected by the registries held by our nephrology colleagues from France [Réseau Epidémiologie et Information en Néphrologie (REIN)], and Flanders, the Dutch speaking part of Belgium [Nederlandstalige Belgische Vereniging voor Nefrologie (NBVN)].

## RESULTS

### Baseline characteristics of the incident ESKD patients

Our patient population is very heterogenous: out of 497 patients, 161 (32%) are WE, 104 (21%) are EE, 128 (26%) are NA, and 104 (21%) are SSA (Table 1). The male/female ratio is 1.5:1 in all groups. Among EE and SSA, most were recent immigrants (including 25% of asylum seekers), whereas NA included both recent and established immigrants, and WE were predominantly native-born.

Groups significantly differ according to their age profile and comorbidities (Fig. 1). The WE patients are significantly older than NA (*P* < .001), EE (*P* < .001), and SSA (*P* < .001). The age difference between EE and SSA is non-significant. The median CCI for WE is 4 while it is 3 for the other sub-groups. The WE population has a significantly higher CCI than the other groups (*P* = .01). Primary renal diseases are detailed in Table 1.

The median time of pre-dialysis follow-up by a nephrology team is 7 months in the WE patient group [interquartile range (IQR) = 36 months], whereas it is shortened to <2 months for all other groups: EE (IQR = 17.5 (months), NA (IQR = 16 months), and SSA (IQR = 12 months) (*P* = .07).

### Treatment modalities and outcome

The HD/PD ratio is 3:1 among WE and SSA, and 4:1 among NA and EE (Table 1). The time to reach 25% cumulative incidence of transplantation is, respectively 91 months, 127 months and 61 months for EE, NA, SSA while it is never reached for the WE group. The actuarial median survival time is lower in the WE group (49 months) compared with 75 months (EE), 75 months (NA) and 98 months (SSA) (*P* < .0001). Most recent figures show that 14% of Western Europeans remain on dialysis, 45% are deceased, and 15% have received a transplant. In parallel, 17% of SSA patients are deceased and 28% have received a transplant (Table 1).

### Univariate and multivariate survival analyses

**Figure 2:**
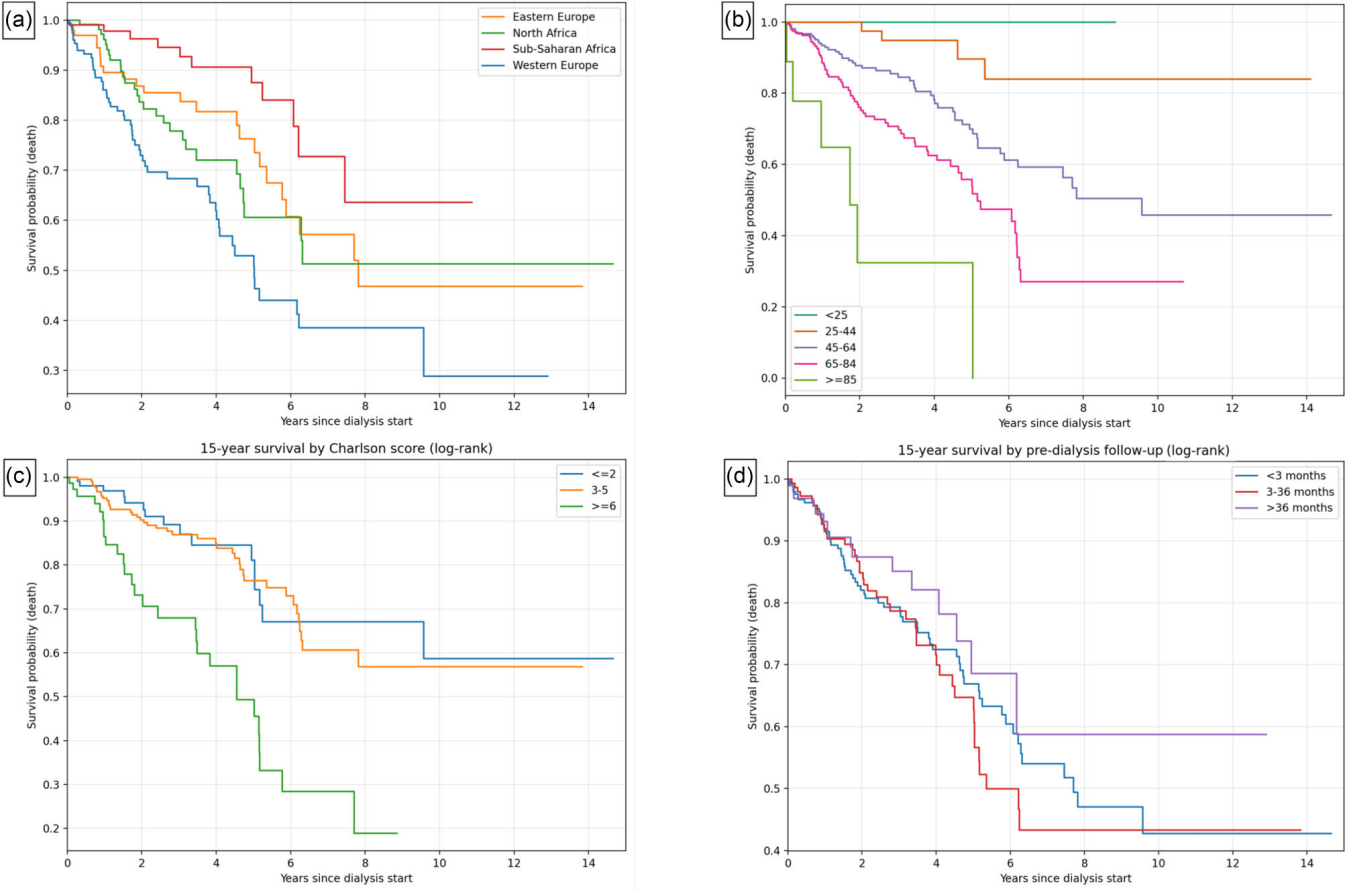
Fifteen-year survival variations by origin, age, Charlson comorbidity score, and pre-dialysis follow-up (Kaplan–Meier, log-rank). (a) Fifteen-year survival differs by ethnic origin (log-rank: *χ*² = 25.14, *P* < .001). (b) Fifteen-year survival decreases with increasing age (log-rank: *χ*² = 47.04; *P* < .001). (c) Higher comorbidity burden is associated with lower 15-year survival (log-rank: *χ*² = 27.89; *P* < .001). (d) Pre-dialysis nephrology follow-up does not influence 15-year survival (log-rank: *χ*² = 1.80; *P* = .406).

Kaplan–Meier analyses with log-rank testing showed significant differences in survival according to ethnic origin (log-rank: *χ*² = 32.6; *P* < .001) (Fig. 2a), age group (log-rank: *χ*² = 63.7; *P* < .001) (Fig. 2b), and CCI (log-rank: *χ*² = 20.7; *P* < .001) (Fig. 2c). No association was found with gender, dialysis modality, primary renal disease, or pre-dialysis nephrological follow-up (Fig. 2d). Using Cox regression modelling with ethnic origin as the single covariate and the patients from WE as the reference group, the estimated HRs for the other groups are: EE HR = 0.50 (95%CI : 0.33–0.76, *P* = .001), NA HR = 0.54 (95%CI : 0.36–0.80, *P* = .002), and SSA HR = 0.28 (95%CI : 0.16–0.46, *P* < .0001).

As all the covariates we considered are susceptible to impact on survival even if only three had a statistically significant effect in our dataset, we assessed the association between ethnic origin using a Cox proportional hazards model with a full adjustment. The ethnic origin keeps a significant impact on survival, driven by the difference between WE and SSA with a HR = 0.30 (95% CI 0.16–0.55, *P* = .0001). The other significant factors are age and the Charlson index. The full model is presented in Table 2. If we try to identify a more parsimonious model thanks to a stepwise backward selection of the covariates, we remain with three significant covariates (age, Charlson index, and ethnic origin). The hazard ratio estimates for ethnic origin remain stable between the full model and the restricted model.

**Table 2:** Multivariable Cox proportional hazards regression analysis of factors associated with mortality.

	Full model		Backward selection	
	HR (95%CI)	*P*	HR (95% CI)	*P*
Ethnic origin (ref. WE)				
EE	**0.72 (0.42–1.24)**	**.24**	**0.79 (0.47–1.33)**	**.38**
NA	**0.82 (0.50–1.33)**	**.42**	**0.79 (0.49–1.27)**	**.32**
SSA	**0.30 (0.16–0.56)**	**.0001**	**0.36 (0.20–0.65)**	**.0007**
Age (logarithmic scale)	7.07 (2.72–18.38)	<.0001	6.07 (2.33–15.84)	.0002
Sex (ref. = male)				
Female	0.69 (0.47–1.01)	.06		
Charlson (ref. score ≥6)				
Score ≤2	0.52 (0.29–0.94)	.03	0.43 (0.25–0.76)	.004
Score between 3 and 5	0.46 (0.29–0.73)	.001	0.40 (0.26–0.63)	<.0001
Length of follow-up pre-dialysis (ref. <3 months)				
Between 3 and 36 months	0.88 (0.58–1.33)	.54	-	-
>36 months	0.67 (0.38–1.18)	.17		
Type of dialysis (ref. PD)				
Haemodialysis	1.23 (0.79–1.92)	.36	-	-
Primary renal disease (ref. renal vascular disease)				
Type II diabetes	0.88 (0.54–1.44)	.62	-	-
Other	0.72 (0.42–1.23)	.22		
Unknown	1.86 (1.01–3.43)	.05		

Bold values indicate statistically significant results (*P* < .05).

### Kidney transplantation access by ethnic origin, a competing-risk analysis

**Figure 3:**
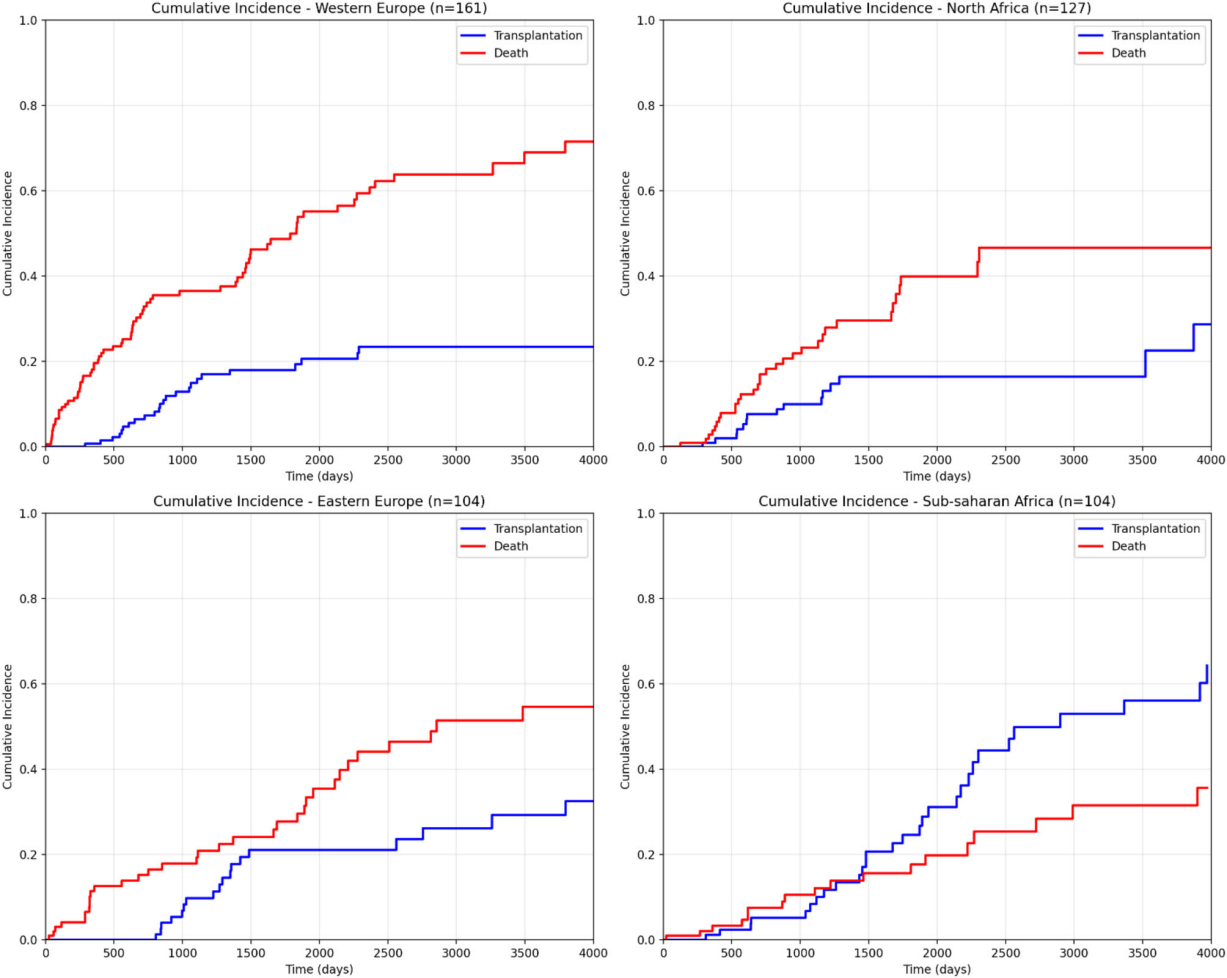
Cumulative incidence of kidney transplantation by ethnic origin using the Fine–Gray competing risks model (death as competing event). CIF were estimated using the Fine–Gray competing risks model, with death considered as the competing event. WE patients served as the reference group. SSA patients demonstrated a significantly higher probability of transplantation (SHR 2.13, 95% CI: 1.27–3.58, *P* = .004), whereas EE (SHR 1.13, 95% CI: 0.62–2.06, *P* = .690) and NA patients (SHR 0.92, 95% CI: 0.48–1.77, *P* = .810) showed no significant differences compared with Western Europeans.

The cumulative incidence analysis of kidney transplantation over 15 years (2010–2024) using the Fine–Gray competing risks model with death as the competing event revealed notable differences according to patients' geographic origin (Fig. 3). Patients from SSA demonstrated the highest cumulative incidence of transplantation, reaching ∼56% at 10 years, followed by EE patients with 29% at 10 years. WE patients (reference group) achieved ∼23% at 10 years, while NA patients presented the lowest incidence at around 22% at 10 years. These cumulative incidence differences at 10 years were corroborated by crude transplantation rates, with the highest rate observed in SSA patients (27.9%) and the lowest in NA patients (11.7%). CIF for transplantation, with death as a competing event, showed marked between-group differences. At 10 years, the CIF was higher in SSA patients than in WE (0.56 vs 0.23) and higher than in the three other groups combined; overall differences in CIF were significant by Gray’s test (*χ*² = 10.33; degrees of freedom = 3; *P* = .02). Analysis of SHR using WE patients as the reference group confirmed these observations. The results of the multivariable Fine–Gray competing-risk model are presented in Table 3. SSA patients had a sub-distribution hazard ratio of 2.07 (95% CI: 1.22–3.52, *P* = .007), indicating a significantly higher hazard of transplant access compared to Caucasian patients. By contrast, EE patients showed no statistically significant difference with an SHR of 1.10 (95% CI: 0.60–2.02, *P* = .77), and NA patients also demonstrated comparable probability to WE with an SHR of 0.90 (95% CI: 0.46–1.74, *P* = .75). These results indicate that, from an univariate association only, SSA patients have a hazard of accessing transplantation 2-fold higher compared with WE patients, even after adjustment for the competing risk of death. However, if we adjust for age, Charlson score and duration of follow-up in the pre-dialysis setting (covariates chosen for being susceptible to impact on access to transplantation, on survival and correlated to the ethnic origin), the sub-hazard ratio estimates are modified and we do not identify any more a statistically significant association between origin ethnic and access to transplantation (borderline significance, *P* = .07). This suggests that the univariate association is confounded by the younger age of the SSA patients.

**Table 3:** Multivariable Fine–Gray competing-risk model for access to kidney transplantation.

	Univariate model		Adjusted model	
	HR (95%CI)	*P*	HR (95% CI)	*P*
Ethnic origin (ref: WE)				
EE	**1.10 (0.60–2.02)**	**.77**	**0.79 (0.35–1.75)**	**.55**
NA	**0.90 (0.46–1.74)**	**.75**	**0.78 (0.33–1.84)**	**.57**
SSA	**2.07 (1.22–3.52)**	**.007**	**1.89 (0.95–3.73)**	**.07**
Age (logarithmic scale)	-	-	0.22 (0.12–0.39)	<.0001
Length of follow-up pre-dialysis (ref. <3 months)				
Between 3 and 36 months	-	-	1.36 (0.79–2.35)	.27
>36 months			1.61 (0.83–3.12)	.16
Charlson (ref. score ≥6)				
Score ≤2	-	-	1.72 (0.78–3.77)	.18
Score between 3 and 5			1.35 (0.66–2.77)	.42

Bold values indicate statistically significant results (*P* < .05).

## DISCUSSION

### Generalities

Up to 1 billion people worldwide suffer from CKD consisting of 2/3 of undiagnosed cases [1, 2, 11]. In the CKD population, the mortality is substantial, and the leading cause of hospital visits and costs is due to the CKD [11].

Recently, the CaReMe (CArdioRenal and MEtabolic) CKD study [11] including patients from 11 Western countries (including Belgium), and the study of Bello *et al*. [12], including 160 countries, reached unprecedented multinational compilations of patients worldwide, enabling the study of a total population with greater ethnic, and socioeconomic variety. Despite valuable real-world data about ESKD prevalence and patient characteristics, the extrapolation of these results to populations of different races or backgrounds (i.e. immigrants) is unknown as these studies’ data still coming mostly from high-income countries. In these studies, the part of immigrants is unknown and the participation of African countries, for instance, is poor.

Smaller-scaled observational studies have specifically examined the characteristics of patients of different ethnicities (i.e. black ethnicity). Few Western observational studies reported a possible survival advantage on dialysis to the black race [13–21]. This advantage is attributed to a favourable inflammation and nutritional status, including higher muscle mass, a lower incidence of cardiovascular disease, and more kidney-limited diseases in black patients. However, except for the study by van den Beukel *et al*. [18], the role of direct immigrants in these populations is unknown.

To current knowledge, only one low-income country can offer RRT to >50% of medically suitable patients [12]. Fewer than half of low-income countries provide public funding for RRT. Similarly, neither PD as the initial dialysis modality nor kidney transplantation is available in ∼80% of patients from low-income countries [12]. Such inequality in terms of RRT access, directly linked to social inequalities worldwide, is likely to increase health emigration to Europe, where dialysis and transplantation are more accessible.

Beyond global disparities in access to KRT, several European studies have shown that migrant populations face specific inequities in nephrology care. Migrants are more frequently referred late to nephrology, initiate dialysis more often in emergency settings, and rely more frequently on temporary vascular access, with most being unaware of their CKD at arrival [6].

In the field of transplantation, first-generation immigrants born outside the European Union have significantly reduced access to living-donor kidney transplantation, despite similar medical eligibility, while access to deceased-donor transplantation does not differ according to migratory status [7].

These disparities are mainly driven by language barriers, cultural factors, administrative constraints, financial insecurity, and insufficient access to adapted educational programmes [8].

### Socioeconomic profile of our immigrant ESKD patients

The 1951 Convention and the 1967 Protocol related to the Status of Refugees, documents were created by the United Nations and ratified by 145 Member States, Belgium being one of them. These documents define the term ‘refugee’ and set out the rights of uprooted people, as well as the legal obligations of States to ensure their protection. The fundamental principle is non-refoulement, according to which a refugee should not be sent back to a country where his/her life or freedom is seriously threatened. This is typically the case of our immigrant ESKD patients arriving in Brussels and taken care by FEDASIL. They are fleeing war (e.g. Balkans, Ukraine) or precarious living conditions in their country of origin (SSA). As they do not have any health insurance fund from their country of origin and are without any legal residence permit in Belgium, they cannot adhere to any health insurance process. In Belgium, chronic dialysis is fully reimbursed for asylum seekers through FEDASIL and for undocumented migrants through the legally mandated ‘Urgent Medical Aid’ system administered by local Public Centres for Social Welfare (CPAS). This financial framework allows equitable access to dialysis regardless of administrative status, but is not uniformly implemented across European countries. Usually, the medical team of FEDASIL will directly contact our nephrology department or the emergency department of our hospital as soon as ESKD is detected on a routine biological test. As a public hospital, the acceptance of such patients is a priority for our social workers. As soon as the need for dialysis is confirmed, as well as hospitalization for ESKD management, they will apply for an ‘Urgent Medical Aid’ procedure to a Public Centre for Social Welfare (CPAS) in Brussels city or municipality around.

The present medical context explains why the median time period of nephrological follow-up before starting dialysis (in months) is significantly different between groups. Our WE patients are usually taken care in consultation for a long time before eventually entering dialysis.

### Basic demographics

Compared with the NBVN and REIN registries, the age profiles of the WE and NA groups are similar (#70 years old) [22]. In our study, EE and SSA immigrants are significantly younger than WE and NA at the start of dialysis, having thus fewer comorbidities overall (Fig. 1).

In Belgium, the two most common causes of ESKD remain vascular nephropathy and diabetic kidney disease. Notably, in Africa, glomerulonephritis would represent the second leading ESKD aetiology [23]. In our cohort, there is a high proportion of ESKD of unknown aetiology in immigrants, and a lack of ESKD attributed to glomerulonephritis in SSA. Many of them come to us with terminal diseases and without any medical history, so kidney biopsy is often unfeasible.

### Treatment gap: emergency dialysis in immigrant populations

Early evaluation by the nephrologist facilitates patient education about RRT modalities, provide more time to make an informed choice about the modality of dialysis, and allows timely placement of permanent vascular access, which is associated with better dialysis performances and fewer complications [24, 25]. Late evaluation is associated with a higher risk for unplanned first dialysis, complications, hospital costs, longer duration of hospitalization in the first 3 months of dialysis, and worse survival (particularly for diabetic and black patients) [26].

According to the REIN registry, fewer French patients would be treated in emergency each year since 2012 [22]. Our study has shown that WE, mostly native Belgians, are usually dialysed after a median nephrological follow-up of 8 months, whereas the other groups are dialysed within the first month, often admitted on an emergency basis, without any previous clinical management by a nephrologist.

### Outpatient dialysis for immigrants: overcoming social barriers

Worldwide, millions of people die of kidney failure each year owing to a lack of access to RRT [27]. Although it remains the utmost costly, HD is the most prevalent form of RRT (around 90%) in low-, middle-, and high-income countries and is expected to increase worldwide in the coming decades [26, 27]. Concurrently, PD is less widely available. In 2018, an estimated 11% of patients receiving long-term dialysis worldwide were treated with PD. Large variations exist between territories, in part determined by governmental policies [30]. According to the REIN registry, PD use decreases in France since 2012 to reach a prevalence of 6%. In Flanders, the use of PD is also down 1.5% since 2011 to reach a prevalence of 7.5%.

We report a higher use of PD as incident treatment modality (25%–30%). Although SSA and EE patients are usually asylum seekers on first dialysis, remaining in refuge centres for days or months. A lack of home facilities, and language barriers should therefore not be considered as contraindications to PD. Information about dialysis techniques is often available even in acute situations, and well-trained nurses enable better use of PD [31, 32].

### Ethnicity predicts survival independent of dialysis modality

Mortality is notably high among dialysis patients, especially in the first 3 months following the initiation of an HD treatment. Approximately 25% of patients on HD die within a year of initiating therapy in high-income countries, and this proportion is even higher in low-income and middle-income countries [33–35]. Some studies have suggested a survival advantage of black ethnicity. Whether this difference would be due to ethnic origin, differences in health system practices, a combination of these factors, or other unrelated factors is unknown [36].

Factors identified in the medical literature that may contribute to the better survival of African or Afro-descendant patients on dialysis include: a higher prevalence of kidney diseases confined to the organ rather than systemic conditions, leading to lower cardiovascular mortality [13, 37, 38]; a lower burden of systemic atherosclerosis and cardiovascular comorbidities [13, 37, 38]; genetic influences such as APOL1 variants, associated with more localized renal disease and reduced atherosclerosis [37]; a lower likelihood of discontinuing dialysis after severe events [39]; more favourable nutritional and inflammatory profiles [40]; a selection effect at dialysis initiation, with non-African patients often presenting with greater comorbidity [41]; and an age-dependent effect, with the survival advantage particularly marked in patients over 50 [42, 43, 17]. Together, these factors could partially explain the paradox of improved survival observed in African and Afro-descendant patients undergoing dialysis in high-income countries.

Our survival analysis confirms ethnic origin as a significant predictor of mortality, alongside age and comorbidity burden, as shown by Kaplan–Meier log-rank analyses and confirmed in Cox regression. Notably, dialysis modality, pre-dialysis nephrology follow-up duration (late referral), and primary renal disease showed no association with survival outcomes.

The lack of statistical significance of late referral in the adjusted model should not be interpreted as absence of clinical relevance; multicollinearity with age and comorbidity likely attenuated its independent contribution. Overall, the apparent effect of ethnic group most likely reflects demographic and clinical profiles rather than ethnicity as an intrinsic prognostic factor, supporting the hypothesis that demographic and clinical factors rather than healthcare access patterns primarily drive survival differences in our cohort.

### Transplant access: demographic advantages and bureaucratic delays

Kidney transplantation provides the best outcomes at the lowest cost [12]. Over the last 10 years, the French median time to transplantation was 19 months, (including pre-emptive transplantation), and the transplantation rate was 3.5% although highly variable according to the age profile [44]. In the same period, in Flanders, 8.5% of all ESKD patients would be transplanted.

**Figure 4:**
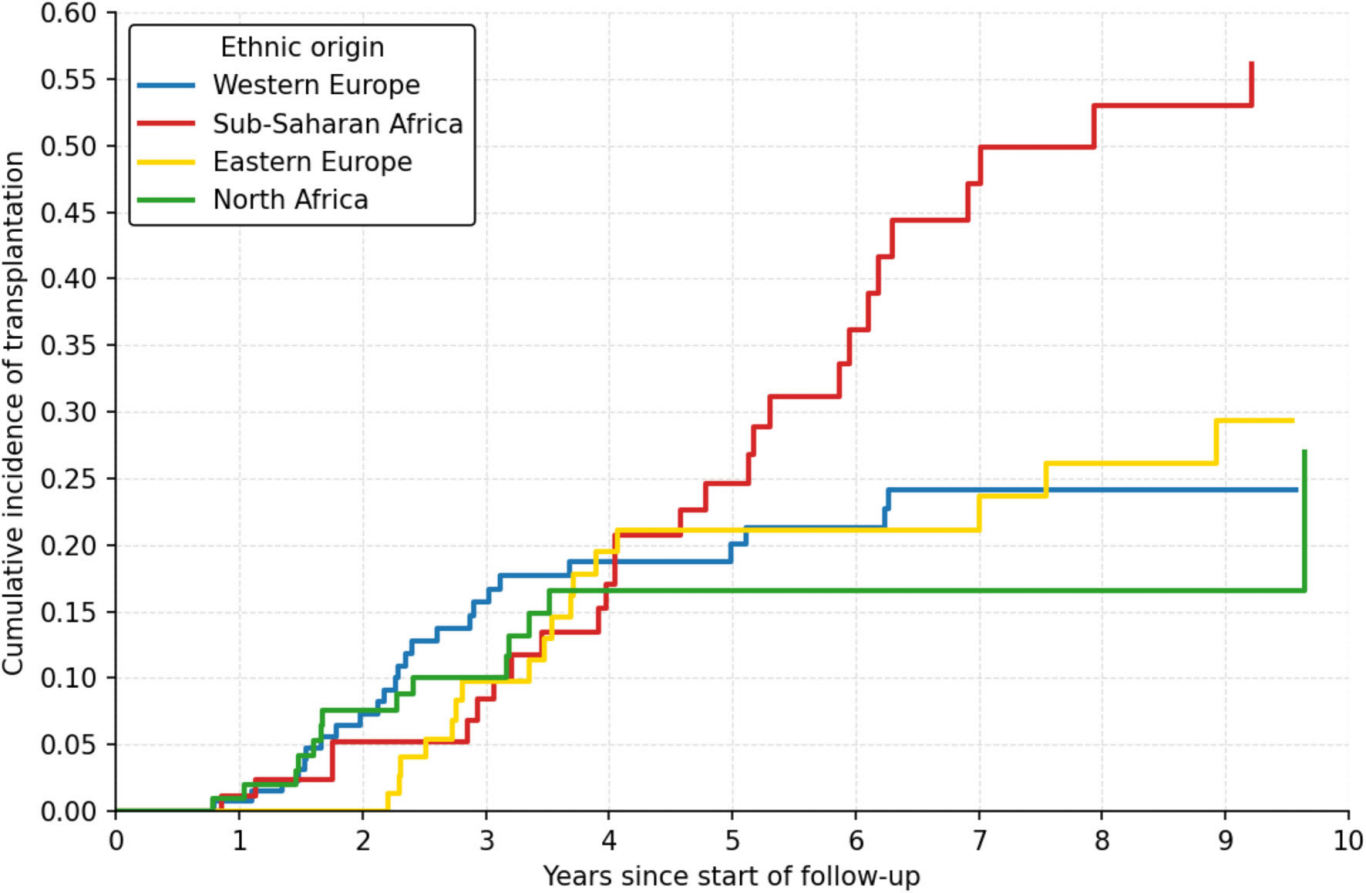
Highest cumulative incidence of kidney transplantation in SSA patients demonstrated by a competing risks analysis. Cumulative incidence of transplantation by ethnic origin illustrated by non-parametric CIF and group comparison using the Fine–Gray model with death as competing risk (Gray test: *χ*² = 22.49; degrees of freedom = 3; *P* < .001).

Our 15-year competing-risk analysis reveals striking differences in transplantation access by ethnic origin (Figs 3 and 4). SSA patients demonstrated the highest cumulative incidence, reaching 35%–40% at 10 years with a sub-distribution hazard ratio of 2.13 (95% CI: 1.27–3.58, *P* = .004), indicating a 113% higher probability of transplant access compared to WE patients. EE patients achieved 25%–30% cumulative incidence at 10 years, while WE and NA patients showed comparable rates of 20%–25% and 15%–20% respectively.

These findings contrast with multiple European and North American studies reporting reduced access to transplantation among SSA patients, including lower waitlisting rates, prolonged waiting times, and reduced transplantation probabilities even after adjustment for clinical and organizational factors. These disparities have been attributed to late referral, socioeconomic barriers, limited access to pre-emptive or living-donor transplantation, and structural inequities [41, 45–49].

In our centre, however, the higher likelihood of transplantation among SSA patients appears largely explained by their younger age and lower comorbidity burden, which are major eligibility criteria in Belgian transplant programmes. Despite often prolonged administrative delays related to legal regularization procedures, transplant access remained favourable in this group.

This divergence from the broader international literature supports one of the key messages of our study: supported by a strong collaboration with social support organizations, access to kidney transplantation appears equitable and guided primarily by objective clinical criteria rather than sociodemographic characteristics. Such organizational features may help mitigate the disparities usually reported among socially vulnerable migrant populations.

## STRENGHTS AND WEAKNESSES

Although monocentric, our study provides original data on a population largely composed of refugees and undocumented immigrants, which is absent from current nephrology literature. This single-centre design was unavoidable, as our hospital is one of the few centres in Belgium combining a very high proportion of recent immigrants, asylum seekers, and undocumented patients within a large public university hospital framework.

Limitations include the lack of biopsy confirmation for all ESKD aetiologies, potentially affecting diagnostic accuracy, and the absence of socioeconomic data that could have further clarified the role of social disparities in renal care. These limitations reflect the constraints of a retrospective design but do not detract from the study’s originality and representativeness. Because transplantation interrupts follow-up for mortality on dialysis, differential access to transplantation across groups may introduce partially informative censoring and could influence survival comparisons.

## CONCLUSION

Our study demonstrates that high-quality RRT can be successfully delivered to diverse immigrant populations. Our population is characterized by an exceptionally high level of diversity and social vulnerability, possibly offering a glimpse of future European dialysis populations as healthcare migration increases in search of more affordable dialysis care.

Our findings challenge conventional assumptions by showing successful PD implementation (25%–30%) even among asylum seekers with language barriers and inadequate housing.

Survival differences across ethnic groups highlight ethnicity as an independent predictor of outcomes in dialysis patients.

Competing-risk analysis reveals that transplantation access reflects appropriate clinical prioritization, with younger SSA patients achieving superior rates (35%–40% at 10 years) based on medical suitability rather than healthcare disparities.

These results provide evidence-based guidance for healthcare systems managing diverse ESKD populations, demonstrating that immigrant status should not preclude access to optimal therapies and that clinical outcomes are primarily determined by patient characteristics rather than geographic origin.

## ETHICS APPROVAL AND CONSENT TO PARTICIPATE

All methods were carried out in accordance with relevant guidelines and regulations. Ethical approval was obtained from the Ethics Committee of Brugmann University Hospital. The need for participants’ informed consents was waived by the Ethics Committee of Brugmann University Hospital, considering the retrospective and anonymous nature of the study.

## Data Availability

Other datasets generated and/or analysed during the current study are not publicly available but are available from the corresponding author on reasonable request.
